# Higher caseload improves cervical cancer survival in patients treated with brachytherapy

**DOI:** 10.1186/s13014-014-0234-2

**Published:** 2014-10-25

**Authors:** Moon-Sing Lee, Shiang-Jiun Tsai, Ching-Chih Lee, Yu-Chieh Su, Wen-Yen Chiou, Hon-Yi Lin, Shih-Kai Hung

**Affiliations:** Department of Radiation Oncology, Buddhist Dalin Tzu Chi Hospital, 2, Ming Sheng Road, Dalin, Chiayi, Taiwan; Department of Otolaryngology, Buddhist Dalin Tzu Chi Hospital, Chiayi, Taiwan; Department of Hematology Oncology, Buddhist Dalin Tzu Chi Hospital, Chiayi, Taiwan; School of Medicine, Tzu Chi University, Hualien, Taiwan

## Abstract

**Objectives:**

Increased caseload has been associated with better patient outcomes in many areas of health care, including high-risk surgery and cancer treatment. However, such a positive volume vs. outcome relationship has not yet been validated for cervical cancer brachytherapy. The purpose of this study was to examine the relationship between physician caseload and survival rates in cervical cancer treated with brachytherapy using population-based data.

**Methods:**

Between 2005 and 2010, a total of 818 patients were identified using the Taiwan National Health Insurance Research Database. Multivariate analysis using a Cox proportional hazards model and propensity scores was used to assess the relationship between 5-year survival rates and physician caseloads.

**Results:**

As the caseload of individual physicians increased, unadjusted 5-year survival rates increased (*P* = 0.005). Using a Cox proportional hazard model, patients treated by high-volume physicians had better survival rates (*P* = 0.03), after adjusting for comorbidities, hospital type, and treatment modality. When analyzed by propensity score, the adjusted 5-year survival rate differed significantly between patients treated by high/medium-volume physicians vs. patients treated by low/medium-volume physicians (60% vs. 54%, respectively; *P* = 0.04).

**Conclusions:**

Provider caseload affected survival rates in cervical cancer patients treated with brachytherapy. Both Cox proportional hazard model analysis and propensity scores showed association between high/medium volume physicians and improved survival.

## Introduction

Cervical cancer remains the most important cause of cancer death in women from Taiwan with an age-adjusted incidence of 26.2 per one hundred thousand women [1,2]. The combination of chemotherapy administered concurrently with radiotherapy shows survival benefit in patients with bulky and locally advanced cervical cancer [3]. However, dose is related to both local control of tumor growth and overall survival. The risk of tissue toxicity currently limits the external radiation dose that can be safely delivered [4]. Thus, brachytherapy is often combined with external beam radiotherapy in cervical cancer patients to prevent damage to surrounding normal tissues.

Brachytherapy is a technically demanding process. The “practice makes perfect” hypothesis may be valid for such a procedure, in that increased experience improves patient outcomes. The association between increased caseload and improved patient outcomes has been reported for both high-risk surgery and cancer treatment [1,2]. Positive correlations between improved treatment outcomes and increased caseload volume have been documented for nasopharyngeal cancer, breast cancer, oral cancer, and esophageal cancer [2,5-7]. However, such a positive volume-outcome relationship has not been established for cervical cancer brachytherapy. The purpose of this study was to examine the relationship between physician caseload and survival rates in cervical cancer patients treated with brachytherapy, using population-based data.

## Materials and methods

### Ethics statement

The study protocol was approved by the Buddhist Dalin Tzu Chi General Hospital Institutional Review Boards. The institutional review board waived the need for written informed consent from the participants because the data analyzed consisted of anonymous secondary data released to the public for research.

### Patients and study design

Between 2005 to 2010, data from the National Health Insurance (NHI) Research Database was used in this study. This data contained all covered medical benefit claims for over 23 million people in Taiwan (approximately 97 percent of the island’s population). All patients with cervical cancer (as defined by International Classification of Disease, Ninth Revision, Clinical Modification codes 180) were included who received radiotherapy or chemoradiotherapy between 2005 and 2007. Patients were excluded who had unclear treatment modalities or incomplete physician data. Finally, 818 patients, treated by 93 radiation oncologists during this 5-year period, were included in our analysis.

Physicians were further stratified by their total patient volumes (using the unique physician identifiers in this database) and by their caseload of cervical cancer patients. The volume category cutoff points (high, medium, and low) were determined by sorting the 818 patients into three groups (1–11 cases = low caseload), 12–40 cases = medium caseload, and ≧41 cases = high caseload), as previously described [5,8]. The volume category cutoff points were determined by sorting the sample into 3 approximately equal groups, so that each group would have approximately equal numbers of patients. These cervical cancer patients were then linked to death data extracted from the records covering the years between 1996 and 2010.

### Measurements

The key dependent variable of interest was the 5-year survival rate. The key independent variables were the cervical cancer caseloads (low, medium, or high). Other physician characteristics included age (≦40, 41–50, ≧51 years) and gender. Patient characteristics included age, geographic location, treatment modality, severity of disease, enrollee category (EC), and urbanization. The disease severity in each patient was assessed using the modified Charlson comorbidity index score, which has been widely used, in recent years, for risk adjustment in administrative claims data sets [9].

This study used EC as a proxy measure of socioeconomic status, which is an important prognostic factor in cancer patients [10,11]. Patients with cervical cancer were classified into four subgroups: EC 1 (civil servants, full-time, or regular paid personnel with a government affiliation), EC 2 (employees of privately owned institutions), EC 3 (self-employed individuals, other employees, and members of farmers’ or fishermen’s associations), EC 4 (veterans, low-income families, and substitute service draftees), and other [12]. In Taiwan, government affiliated workers have stable job occupation and fixed salary in every month than self-employed. On average, government affiliated workers’ payroll-related amount for the health insurance was highest.

The hospitals were categorized by ownership (public, not-for-profit, or for-profit) and hospital type (medical center, regional hospital, or district hospital).

### Statistical analysis

The SAS statistical package (version 9.2; SAS Institute, Inc., Cary, N.C.) and SPSS (version 21, SPSS Inc., Chicago, IL, USA) was used for data analysis. A two-sided *P* value < 0.05 was used to determine statistical significance.

The cumulative 5-year survival rates and the survival curves for each group were compared by the log-rank test. Survival was measured from the time of cervical cancer diagnosis to the time of death. Cox proportional regression model and survival analysis using propensity score stratification were used to compare outcomes between different groups based on caseload.

### Cox proportional hazards model

The Cox proportional regression model was used to evaluate the effect of caseload on survival rate after adjusting for hospital type, surgeon characteristics, and patient demographics.

### Propensity score

Propensity analysis was used to reduce the effect of selection bias on our hypothesis, as described by Rosenbaum and Rubin [13-15]. Propensity score stratification replaced the many confounding factors that might be present in an observational study with such a variety of factors. To calculate the propensity score in this study, patient characteristics were entered into a logistic regression model that predicted selection for high/medium-volume surgeons. These patient characteristics included the year in which the patient was diagnosed, their age, gender, Charlson comorbidity index score, geographic area of residence, enrollee category, and treatment modality. The study population was then divided into five discrete strata based on propensity score. The effect of caseload assignment on 5-year survival rates was analyzed within each quintile. The Mantel-Haenszel odds ratio was calculated in addition to the Cochran-Mantel-Haenszel χ^2^ statistic.

## Results

A total of 346 out of 818 patients (42%) died after undergoing treatment between 2005 and 2007. A total of 93 radiation oncologists were included in the analysis. The characteristics of the physicians and patients are summarized in Tables 1 and 2. Patients in the low-volume physician group were more likely to undergo radiotherapy, reside in Southern and Eastern Taiwan, and have higher comorbidity score, than their counterparts in other groups. There were 74 radiation oncologists (80%) in the low-volume group, 14 physicians (15%) in the medium-volume group, and five (5%) physicians in the high-volume group. The mean age of all physicians was 41 ± 6 years. There was no significant difference among physicians who comprised these three caseload groups with regards to age (*P* = 0.90).

**Table 1:** **Patients characteristics according to caseload (n = 818)**

	**Cervical cancer caseload group**			
**Variable**	**Low**	**Medium**	**High**	***P*** **-value**
	**(1–11)**	**(12–40)**	**(41–78)**	
	**(n = 280)**	**(n = 262)**	**(n = 276)**	
**Age**				<0.001
25-44 years	23(8.2)	29(11.1)	40(14.5)	
45-54 years	53(18.9)	57(21.8)	74(26.8)	
55-64 years	47(16.8)	33(12.6)	58(21.0)	
65-74 years	76(27.1)	68(26.0)	60(21.7)	
≧75 years	81(28.9)	75(28.6)	44(15.9)	
**Charlson comorbidity index score**				0.009
0	103(36.8)	113(43.1)	139(50.4)	
1-3	109(38.9)	78(29.8)	80(29.0)	
≧4	68(24.3)	71(27.1)	57(20.7)	
**Treatment modality**				0.001
Radiotherapy	122(43.6)	82(31.3)	83(30.1)	
Chemoradiotherapy	158(56.4)	180(68.7)	193(69.9)	
**Geographic location**				<0.001
North	98(35.0)	108(41.2)	57(20.7)	
Central	74(26.4)	84(32.1)	129(46.7)	
Southern and Eastern	108(38.6)	70(26.7)	90(32.6)	
**Enrollee category**				0.89
EC 1-2	60(21.4)	57(21.8)	57(20.7)	
EC 3	109(38.9)	107(40.8)	100(36.2)	
EC 4	57(20.4)	51(19.5)	58(21.9)	
Other	54(19.3)	47(17.9)	61(22.1)	
**Urbanization**				0.14
Urban	66(23.6)	84(32.1)	73(26.4)	
Suburban	133(47.5)	102(38.9)	130(47.1)	
Rural	81(28.9)	76(29.0)	73(26.4)	

Values are given as number (percentage).

**Table 2:** **Physician characteristics (n = 93)**

	**Physician caseload group**			
**Variable**	**Low**	**Medium**	**High**	***P*** **-value**
	**(1–11)**	**(12–40)**	**(41–78)**	
**Total no. of physicians**	74	14	5	
**Age (years)**				0.90
Mean ± SD	42 ± 8	41 ± 6	41 ± 4	
**Gender**				0.71
Male	64(86)	13(92)	4(80)	
Female	10(13)	1(7)	1(20)	
**Caseload**				<0.001
Mean ± SD	3 ± 2	18 ± 7	55 ± 13	

Values are given as number (percentage).

Abbreviation: SD = standard deviation.

### Analysis using a Cox proportional hazards model

**Figure 1:**
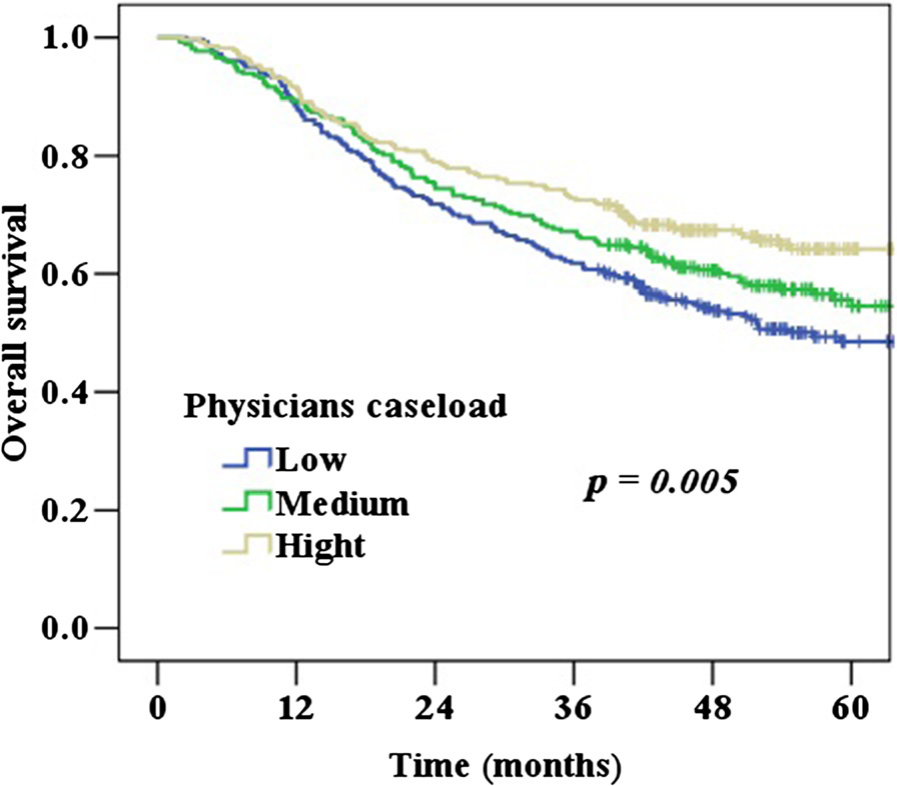
**Cervical cancer survival rates by physician caseload.**

The 5-year survival rates, by physician caseload group, are shown in Figure 1. The 5-year survival rates were 48%, 54%, and 64% for low-, medium-, and high-volume surgeons, respectively (*P* = 0.005). Table 3 shows the adjusted hazard ratios (calculated using the Cox proportional hazards regression model) after adjusting for patient comorbidities, hospital type, and treatment modality. Physicians’ age and 5-year survival have no association (*P* > 0.05). The hazard ratio for age 41–50, and ≧51 during the 5-year follow-up was 0.94 (*P* = 0.70) and 0.88-times (*P* = 0.56) lower than in ≦40 years respectively. The positive association between survival and physician caseload remained statistically significant after multivariate analysis. Patients treated by high-volume physicians had better survival rates (hazard ratio [HR] = 0.69; 95% confidence interval [CI], 0.50-0.97; *P* = 0.03), after adjusting for other factors.

**Table 3:** **Cervical cancer survival rate and adjusted hazard ratios by physician caseload groups and the characteristics of the patients and providers (n = 818)**

**Variable**	**Adjusted hazard ratio**	**95% CI**	***P*** **-value**
**Physician characteristics**			
**Physician volume**			
Low (1–11)	1		
Medium (12–40)	0.90	(0.69-1.19)	0.49
High (41–78)	0.69	(0.50-0.97)	0.03
**Physician age**			
≦40 years	1		
41-50 years	0.94	(0.72-1.24)	0.70
≧51 years	0.88	(0.56-1.36)	0.56
**Hospital characteristics**			
**Hospital ownership**			
Public	1		
Non-for-profit	0.95	(0.72-1.25)	0.74
For-profit	0.98	(0.69-1.40)	0.95
**Hospital level**			
Medical center	1		
Regional hospital	0.70	(0.71-1.26)	0.70
District hospital	1.59	(1.01-2.49)	0.04
**Patient characteristics**			
**Patient age**			
25-44 years	1		
45-54 years	1.26	(0.82-1.92)	0.28
55-64 years	1.11	(0.70-1.74)	0.64
65-74 years	0.96	(0.62-1.48)	0.85
≧75 years	1.23	(078–1.94)	0.36
**Comorbidity index score**			
0	1		
1-3	1.40	(1.07-1.83)	0.01
≧4	2.52	(1.93-3.29)	<0.001
**Treatment modality**			
Chemoradiotherapy	1		
Radiotherapy	1.23	(1.08-1.40)	0.002
**Geographic location**			
North	1		
Central	1.17	(0.85-1.62)	0.32
Southern and Eastern	1.12	(0.81-1.55)	0.47
**Enrollee category**			
Other	1		
EC 1-2	0.92	(0.65-1.30)	0.65
EC 3	1.02	(0.74-1.39)	0.88
EC 4	1.09	(0.77-1.54)	0.61
**Urbanization**			
Urban	1		
Suburban	0.73	(0.56-0.95)	0.02
Rural	0.66	(0.48-0.91)	0.01

95% CI, 95% confidence interval.

### Analysis using propensity scores

Patients were stratified by propensity score and the effect of physician caseload on survival was assessed. The population was stratified into propensity quintiles, as previously described. Table 4 shows the survival rates for caseload groups after stratification. The percentage of patients treated by low-volume physicians decreased from the first propensity quintile to the fifth, as predicted by the propensity model. In each of the five strata, patients treated by high-volume physicians had a higher 5-year survival rate. While controlling for propensity score (with fewer patients dying who were treated by high/medium-volume physicians), the *P* value equaled 0.04 using Cochran-Mantel-Haenszel statistics. This analysis demonstrated a significant difference in survival between patients treated by low vs. high/medium-volume physicians, (adjusted odds ratio = 0.71, 95% CI, 0.51-0.99). The adjusted 5-year survival rates for low vs. high/medium-volume physicians were 54% vs. 60%, respectively (*P* = 0.04).

**Table 4:** **5-year survival rates of cervical patients according to propensity score strata; low-volume vs. high/medium-volume physicians** ^**a**^

**Propensity score stratum**	**Low-volume physician group**			**High/medium-volume physician group**			
	**No.**	**% of stratum**	**Survival rate (%)**	**No.**	**% of stratum**	**Survival rate (%)**	***P*** **-value**
1	112	68	50	51	31	52	0.07
2	84	51	50	80	48	62	0.64
3	50	30	42	114	69	60	0.41
4	19	11	68	145	88	59	0.44
5	15	9	60	148	90	66	0.02
Total	280		54	538		60	0.09
							0.04^b^

^a^Stratum 1 had the strongest propensity for low-volume physician; Stratum 5, for high/medium-volume physicians.

^b^Cochran-Mantel-Haenszel statistics; adjusted odds ratio = 0.71,95% confidence interval = 0.51-0.99.

In summary, cervical cancer patients treated by higher volume physicians showed improved survival. The robustness of this result was demonstrated by two different multivariate analyses, the Cox proportional regression model and stratification by propensity score.

## Discussion

Improved patient outcomes have been correlated with higher caseload volumes. However, there is limited data about the use of brachytherapy in cervical cancer patients. Although the Royal College of Radiologists has made the pragmatic decision to maintain sufficient experience and expertise, they are not backed by any published evidence [16]. We used a Cox proportional hazards model and propensity score to evaluate the relative patient benefit of treatment by high/medium-volume physicians vs. low -volume physicians using cervical cancer brachytherapy. From these results of both forms of multivariate analyses, we found that the 5-year survival rates for brachytherapy patients treated by high/medium -volume physicians were significantly better compared to patients treated by low-volume physicians.

The quality of the risk-adjustment techniques used in analyzing administrative information is an important issue. In the first part of this study, a Cox proportional hazard model was used to compare the effects of high/medium volume versus low volume on survival rates. We found that treatment by high/medium-volume physicians was significantly associated with a lower adjusted hazard ratio for death. Patients treated by high-volume physicians were found to have a 31% lower risk of death after adjusting for comorbidities and other confounding factors. However, there were differences in clinical conditions between caseload groups. In the second part of our series, propensity score was used to stratify patients into five strata with similar propensity score in order to reduce the effect of selection bias on caseload groups [14,15,17]. Patients treated by high/medium-volume physicians were found to have a 6% relative improvement in adjusted 5-year survival rate (*P* = 0.04) compared to those treated by low-volume physicians.

Several hypotheses have been proposed regarding the relationship between caseload volume and outcome. They suggest that increased caseload may help physicians or hospital staff improve their ability to perform treatment procedures, such as planning and manipulation of the radioactive source to target treatment sites, gauze packing, dose calculation or computerized planning. Careful manipulation of the target volume is important for treatment of cervical cancer with brachytherapy. A team that is comfortable with a higher caseload volume may be more adept at administering radiation dosage which improves loco-regional control of cancer and reduces the risk of toxicity to nearby normal organs and tissues.

Although our study revealed some issues that may be useful for policy makers, further research is necessary to identify differences in care and treatment strategies among low-, medium-, and high-volume physicians. In our study, nearly 33% of patients were treated by only five high-volume radiation oncologists. The viewpoints of high-volume physicians may influence the development of effective protocols and clinical practice guidelines. Furthermore, the treatment strategies of high-volume physicians should be analyzed and adopted, throughout the country, to improve survival rates.

Our study had several limitations. First, we could not assess the relationship of caseload to stage, tumor size or local control rate because this information was not available from the database. Although this limitation may influence our results, Begg et al., using a SEER-Medicare linked database, reported that cancer stage and patient age were independent of caseload volume [18]. Though the health system in Taiwan is not complete the same as the one in USA, patient in Taiwan are also free to choose any physician no matter the disease severity, stage or comorbidity. In addition, selective referral bias must also be considered, i.e., healthier patients or patients with early-stage disease may tend to be referred to the high-volume physicians. Although healthier patients tend to be wealthier and they advocate for themselves, the National Health Insurance covered approximately 97 percent of the island’s population and the hospitals are dispersion in Taiwan. The probability for patients’ choice is average. Second, the database does not contain information regarding tobacco use, dietary habits, and body mass index, which might be risk factors for cervical cancer. Taken together, given the robustness of both the evidence and statistical analyses used in this study, these limitations are unlikely to have compromised our results.

In summary, using analyses based on a Cox proportional hazard model and propensity score, we found an association between higher caseload volume and improved survival in cervical cancer patients treated with brachytherapy using population-based data. In conclusion, higher provider caseload affects survival in cervical cancer patients treated with brachytherapy.
